# Identification of selection signals by large-scale whole-genome resequencing of cashmere goats

**DOI:** 10.1038/s41598-017-15516-0

**Published:** 2017-11-09

**Authors:** Xiaokai Li, Rui Su, Wenting Wan, Wenguang Zhang, Huaizhi Jiang, Xian Qiao, Yixing Fan, Yanjun Zhang, Ruijun Wang, Zhihong Liu, Zhiying Wang, Bin Liu, Yuehui Ma, Hongping Zhang, Qianjun Zhao, Tao Zhong, Ran Di, Yu Jiang, Wei Chen, Wen Wang, Yang Dong, Jinquan Li

**Affiliations:** 1 College of Animal Science, Inner Mongolia Agricultural University, Hohhot, Inner Mongolia, 010018 China; 2 Key Laboratory of Animal Genetics, Breeding and Reproduction - Inner Mongolia Autonomous Region, Inner Mongolia Agricultural University, Hohhot, Inner Mongolia, 010018 China; 3 Key Laboratory of Mutton Sheep Genetics and Breeding, Ministry of Agriculture, Inner Mongolia Agricultural University, Hohhot, Inner Mongolia, 010018 China; 4 Engineering Research Center for Goat Genetics and Breeding - Inner Mongolia Autonomous Region, Inner Mongolia Agricultural University, Hohhot, Inner Mongolia, 010018 China; 5 State Key Laboratory of Genetic Resources and Evolution, Kunming Institute of Zoology, Chinese Academy of Sciences, Kunming, Yunnan 650223 China; 6 Center for Ecological and Environmental Sciences, Key Laboratory for Space Bioscience & Biotechnology, Northwestern Polytechnical University, Xi’an, Shaanxi 710072 China; 7 College of Animal Science and Technology, Jilin Agricultural University, Changchun, Jilin, 130118 China; 8 Institute of Animal Husbandry, Academy of Agriculture and Stockbreeding Sciences, Hohhot, Inner Mongolia, 010030 China; 9 The Key Laboratory for Farm Animal Genetic Resources and Utilization of Ministry of Agriculture of China, Institute of Animal Science, Chinese Academy of Agricultural Sciences, Beijing, 100193 China; 10 Farm Animal Genetic Resources Exploration and Innovation Key Laboratory of Sichuan Province, College of Animal Science and Technology, Sichuan Agricultural University, Chengdu, 611130 China; 11 College of Animal Science and Technology, Northwest A&F University, Yangling, 712100 China; 12 College of Biological Big Data, Yunnan Agriculture University, Kunming, Yunnan 650504 China; 13 BGI-Shenzhen, Shenzhen, Guangdong, 518083 China; 14 Yunnan Research Institute for Local Plateau Agriculture and Industry, Kunming, Yunnan 650201 China

## Abstract

Inner Mongolia and Liaoning cashmere goats are two outstanding Chinese multipurpose breeds that adapt well to the semi-arid temperate grassland. These two breeds are characterized by their soft cashmere fibers, thus making them great models to identify genomic regions that are associated with cashmere fiber traits. Whole-genome sequencing of 70 cashmere goats produced more than 5.52 million single-nucleotide polymorphisms and 710,600 short insertions and deletions. Further analysis of these genetic variants showed some population-specific molecular markers for the two cashmere goat breeds that are otherwise phenotypically similar. By analyzing *F*_ST_ and θ_π_ outlier values, we identified 135 genomic regions that were associated with cashmere fiber traits within the cashmere goat populations. These selected genomic regions contained genes, which are potential involved in the production of cashmere fiber, such as *FGF5*, *SGK3*, *IGFBP7*, *OXTR*, and *ROCK1*. Gene ontology enrichment analysis of identified short insertions and deletions also showed enrichment in keratinocyte differentiation and epidermal cell differentiation. These findings demonstrate that this genomic resource will facilitate the breeding of cashmere goat and other *Capra* species in future.

## Introduction

**Figure 1:**
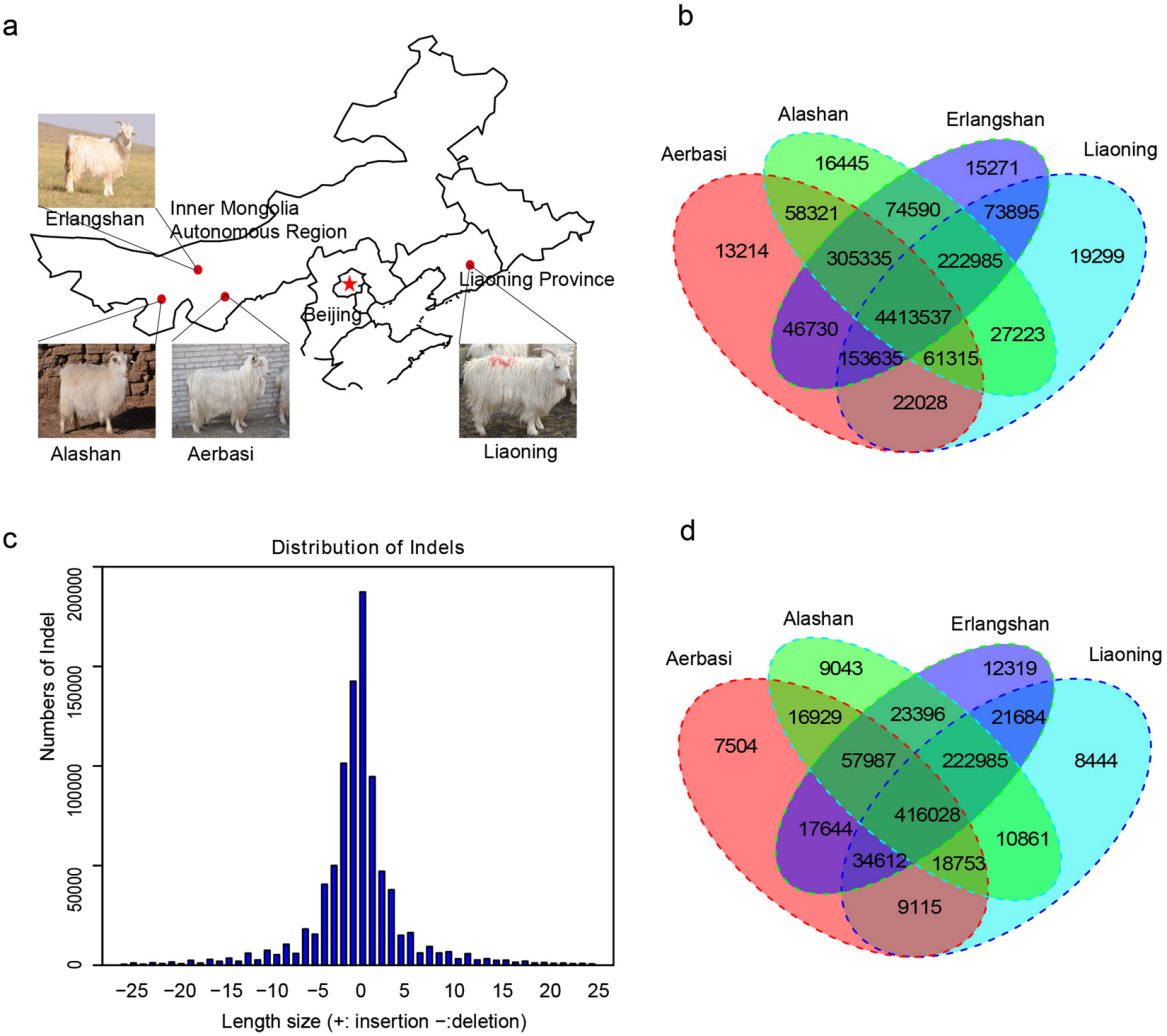
Summary of cashmere goats. (**a**) Geographic map indicating the distribution of the cashmere goats sampled in this study (Photographs were taken by Rui Su and Xiaokai Li). Each red dot represents the location of sampling. The map was generated using the ‘ggmap’ package in R (version 3.4.1) (https://cran.r-project.org/)^60,61^ and trimmed by Adobe Photoshop CS6 (http://www.adobe.com/). (**b**) Venn diagram of SNVs shows the overlap and population-specific identified SNVs among four cashmere goat populations. (**c**) Distribution of InDels. The length of each bar represents the number of InDels. (**d**) Venn diagram of InDels show the common and population-specific genetic variants among four cashmere goat populations.

Cashmere goat grows an outer coat of coarse hairs from its primary hair follicles and an inner coat of fine wool from its secondary hair follicles. This special fine wool fiber is known as cashmere wool or cashmere^1,2^. It is finer and softer than sheep’s wool, and contributes high economic values to the textile industry and impoverished remote areas^3,4^. China is a major cashmere producer in the world, and has rich native cashmere goat genetic resources. In 2012, China supplied about 70% (18 thousand tons) of cashmere wool to the world market^5^. The Inner Mongolia (three subtypes: Alashan, Aerbasi, and Erlangshan^6^) and Liaoning cashmere goats are two native breeds characterized by the thin cashmere fiber diameter and high yield of cashmere wool (Fig. 1a)^7^. For this reason, great research interest has been dedicated to finding new goat breed that produces finer and higher yield of cashmere wool^8–13^.

Over the past decade, the next-generation sequencing technology has markedly facilitated the genetic studies of complex traits in domestic animal^14–16^. This technology has been used to reveal natural and artificial selection footprint in many species, such as pig^17,18^, sheep^19^, dog^20,21^, and so on. With whole-genome resequencing of different sheep breeds, researchers have provided a comprehensive insight into the genetic basis of adaptive variation of sheep in different environment. For example, genes *OAR22_18929579-A, IFNGR2, MAPK4, NOX4, SLC2A4* and *PDK1* showed an apparent geographic pattern and significant correlations with climatic variation^19,22^. Based on the draft goat genome assembly CHIR_1.0 and CHIR_2.0^23^, several preliminary studies have attempted to explore the economic and adaptive traits in different goat breeds using the whole-genome resequencing strategy^24–26^. Using parallel sequencing of pooled DNA from eight goat breeds, Wang *et al*. identified several genomic regions under strong selection that were associated with body size (e.g. *TBX15, DGCR8, CDC25A*, and *RDH16*), cashmere fiber (e.g. *LHX2, FGF9*, and *WNT2*), and coat color (e.g. *ASIP, KITLG*, and *HTT*)^10^. Guan *et al*. identified some candidate genes (e.g. *FGF5*) for improving fiber traits using the whole genome sequence of six cashmere goats and six non-cashmere goats^26^. Despite these useful findings, the sample sizes of these studies were invariably small to elucidate the genetic basis of cashmere fiber trait. Furthermore, these studies did not include Inner Mongolia and Liaoning cashmere goats in their samples, which may miss important genetic information with regard to cashmere goat traits.

Here, we report the whole-genome resequencing of 70 cashmere goats from the Inner Mongolia and Liaoning regions. Analyses of the genetic variants identified population-specific molecular markers and candidate genomic regions under selective sweep that were related to cashmere traits. This genetic resource will not only help with future genome-wide association studies, but also increase the knowledge regarding the genetic architecture of quantitative traits.

## Results and Discussion

### Whole-genome sequencing and genetic variant mapping

A total of 611.67 Gb paired-end DNA sequence data were obtained from 70 female cashmere goats on an Hiseq-2000 platform (Illumina, San Diego, CA, USA). About 534.66 Gb high-quality paired-end reads could be mapped to the latest goat reference genome assembly with a 2.61-fold average coverage (Supplementary Table 1). These data yielded 5,523,823 single-nucleotide polymorphisms (SNPs) and 710,600 short insertions and deletions (Indels) (MAF > 0.5; Fig. 1b and d; Supplementary Table 2). Compare to the dbSNP database (https://ftp.ncbi.nih.gov/snp/organisms/goat_9925/VCF/), about 4,819,577 (87.25) SNPs and 643,205 (90.52%) Indels were novel. The average transition-to-transversion (Ti/Tv) ratio was 2.36 for all cashmere goat samples, which indicated relatively low potential random sequencing errors. This number is comparable to previously reported Ti/Tv ratios for Moroccan goat (2.44) and Dazu black goat (2.33)^25,27^, indicating high accuracy for the identified variants in this study (Supplementary Table 3). The density of SNPs along each chromosome (except X chromosomes) was proportional to the chromosome length (Supplementary Table 2). This result is consistent with the observation that lower proportion of mutant variants could be found on sex chromosomes in goat^28,29^. Besides, the distribution of site depth of SNP was ranged from 23-fold to 7535-fold, with an average depth of 152.70-fold (Supplementary Fig. 1).

We examined the nucleotide diversity and ratio of heterozygous versus homozygous single nucleotide variations (SNVs) among Inner Mongolia and Liaoning cashmere goat. The higher average ratio of heterozygous versus homozygous SNVs was observed in Erlangshan population (Supplementary Table 3). The overall distributions of Inner Mongolia and Liaoning cashmere goat in terms of nucleotide diversity were similar, of which Alashan population showed lower nucleotide diversity (total average nucleotide diversity = 5.31 × 10^−4^) than other populations (Supplementary Table 3 and Supplementary Fig. 2).

About 4,413,537 (79.90%) of identified SNPs were shared among all cashmere goat populations, indicating a high genetic similarity within cashmere goats. This is in line with the report that Inner Mongolia and Liaoning cashmere goats came from a recent common origin^7^. The number of population-specific SNPs was highest in the Liaoning population (19,299 or 0.35%), and was lowest in the Aerbasi population (13,214 or 0.24%) (Fig. 1b **)**. The number of Indels shared among all four populations was 416,028 (58.55%). The numbers of breed-specific Indels ranged from 7504 (1.06%) in the Aerbasi population to 12,319 (1.73%) in the Erlangshan population (Fig. 1d). Compared to Liaoning cashmere goat (19299), Inner Mongolia cashmere goat (529906) have more specific SNPs that may be related to weaker intensive selection breeding.

### Annotation of SNPs and Indels

The proportions of SNPs in the intergenic, intronic, and exonic regions of the genome were 73.72%, 34.00%, and 0.52%, respectively (Table 1). Among all identified SNPs, 28,968 SNPs could cause changes in the coding sequences of 9,621 genes, including 10,606 non-synonymous nucleotide substitutions, 81 stop-codon gain mutations, and 23 stop-codon loss mutations in the cashmere goat genomes (Supplementary Data 1). Enrichment analysis of these genes identified receptor activity related categories, such olfactory receptor activity (600 genes, *P* = 1.01 × 10^−128^), G-protein coupled receptor activity (754 genes, *P* = 2.09 × 10^−94^), transmembrane signaling receptor activity (871 genes, *P* = 2.57 × 10^−75^), transmembrane receptor activity (889 genes, *P* = 2.17 × 10^−72^), and signaling receptor activity (883 genes, *P* = 1.92 × 10^−64^) (Supplementary Data 2 and Supplementary Fig. 3). In addition, enrichment was found in the basic cellular functions, such as the binding of FAD, syntaxin, cytoskeletal protein, metal ion, actin and protein kinase activity.

**Table 1:** Summary and annotation of SNPs in cashmere goat.

Category		Number of InDels	Percent(%)
3′UTR		26325	0.48
5′UTR		5470	0.01
UTR5;UTR3		13	0
Downstream		32477	0.59
Exonic	nonsynonymous SNV	10606	0.52
	stop gain	81	
	stop loss	23	
	synonymous SNV	16895	
	unknown	1363	
Intergenic		3519939	63.72
Intronic		1877966	34.00
NcRNA_exonic		1250	0.02
NcRNA_intronic		2125	0.04
Splicing		52	0
Updtream		28293	0.51
Upstream/Dowstrean		945	0

The identified Indels were 1 bp to 25 bp in length (Fig. 1c), and the total number of deletions is a little larger than the total number of insertions. The frequency of Indels decreased as the sizes of the Indels increased. The proportions of Indels in the intergenic, intronic, and coding sequences of the genome were 63.42%, 34.29%, and 0.09%, respectively (Supplementary Table 4). About 284 Indels (131 deletions and 153 insertions) may result in frame-shift mutations in the coding sequences of 249 genes (Supplementary Data 3). GO annotation of these affected genes revealed enrichment in the biological process terms, such as keratinocyte differentiation (GO: 0030216), epidermal cell differentiation (GO:0009913), and skin development (GO:0043588) (Supplementary Data 4 and Supplementary Fig. 4).

### Population structure analysis

**Figure 2:**
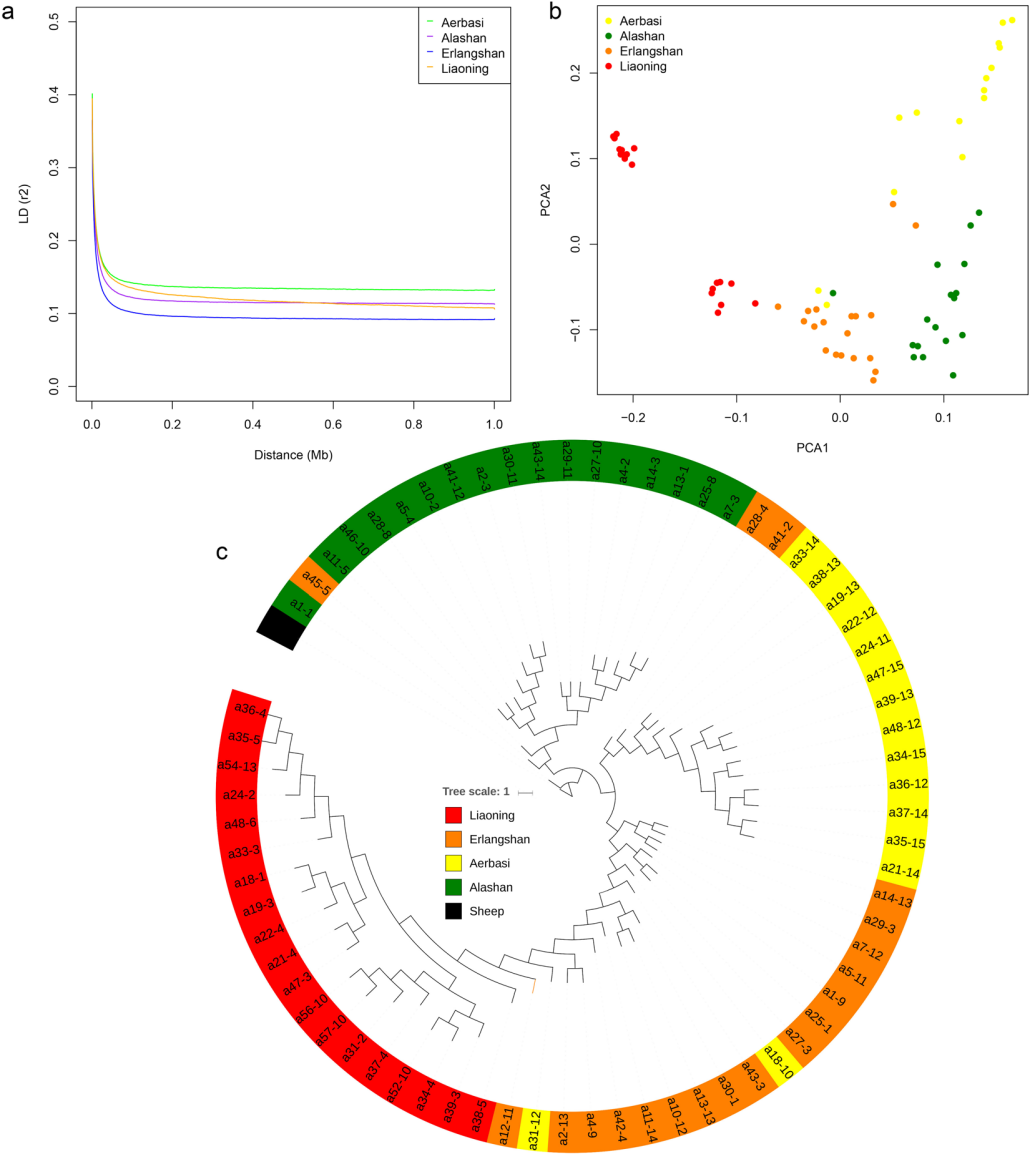
Population genetic relationship analysis. (**a**) LD patterns of cashmere goats (Liaoning and three subtype of Inner Mongolia cashmere goats). (**b**) PCA using thinned SNPs as markers. Each dots are index to samples, and each color represent on population. Most samples cluster together based on their geographic distribution. (**c**) Phylogenetic relationship of cashmere goats. The scale bar represents p distance.

**Figure 3:**
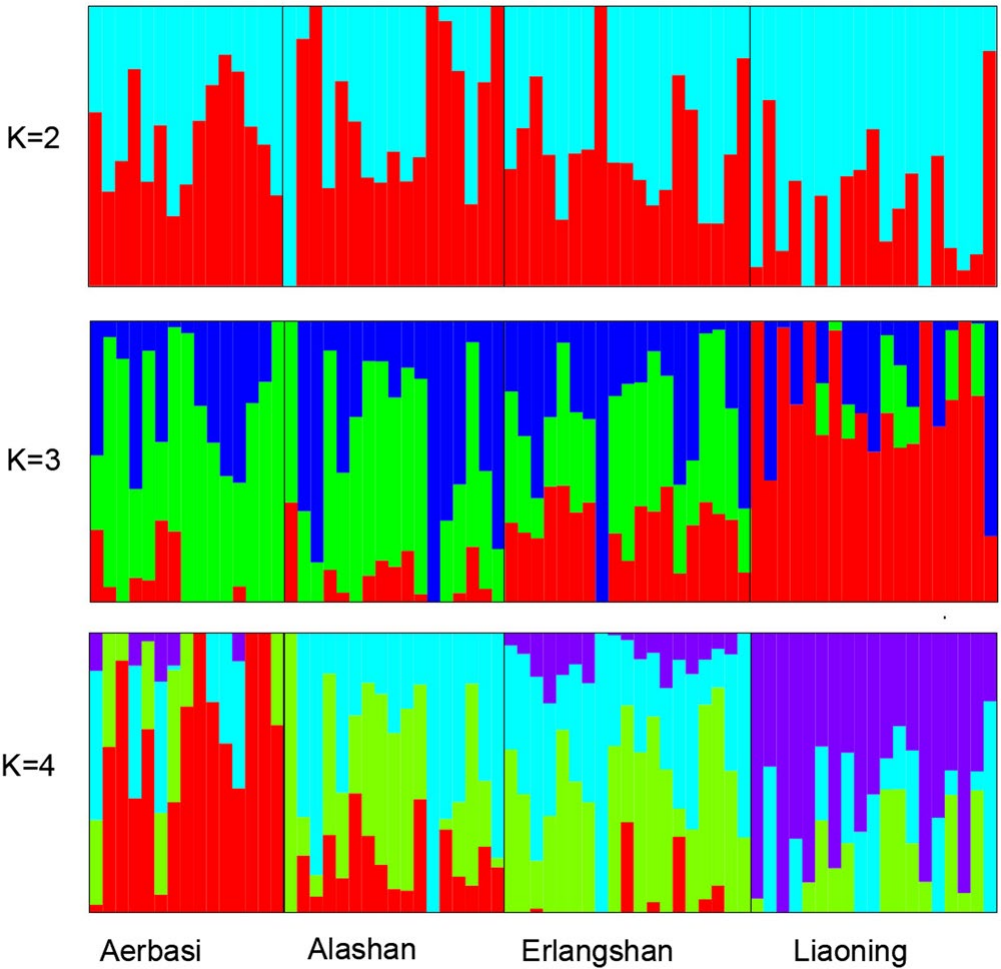
Population structure analysis of cashmere goats using STRUCTURE packages. Each sample is represented by a vertical bar. Enery color represents one ancestral population and the length of each colored seqment in each vertical bar represents the proportion contributed by ancestral populations.

The phylogenetic relationship of the 70 cashmere goats revealed genetically distinct clusters according to their geographic locations (Fig. 2c). This result was confirmed by the principle component analysis (PCA) using thinned genomic SNPs. The first eigenvector distinguished Liaoning cashmere goats from Inner Mongolia cashmere goat, and the second eigenvector distinguished Aerbasi, Alashan and Erlangshan populations (Fig. 2b). The genetic ancestry analysis with STRUCTURE showed that all cashmere goat samples had a mixed ancestry at K = 4 (Fig. 3). Population differentiation index (*F*st) showed that the Aerbasi population had a higher genetic distance (0.11) from the Liaoning cashmere goats, which is consistent with the results of PCA and STRUCTURE (Supplementary Table 5). The linkage disequilibrium (LD) decay rates were similar between Liaoning and Aerbasi populations. The fastest LD decay was observed in the Erlangshan population (Fig. 2a).

### Genome-wide selective sweep signals

**Figure 4:**
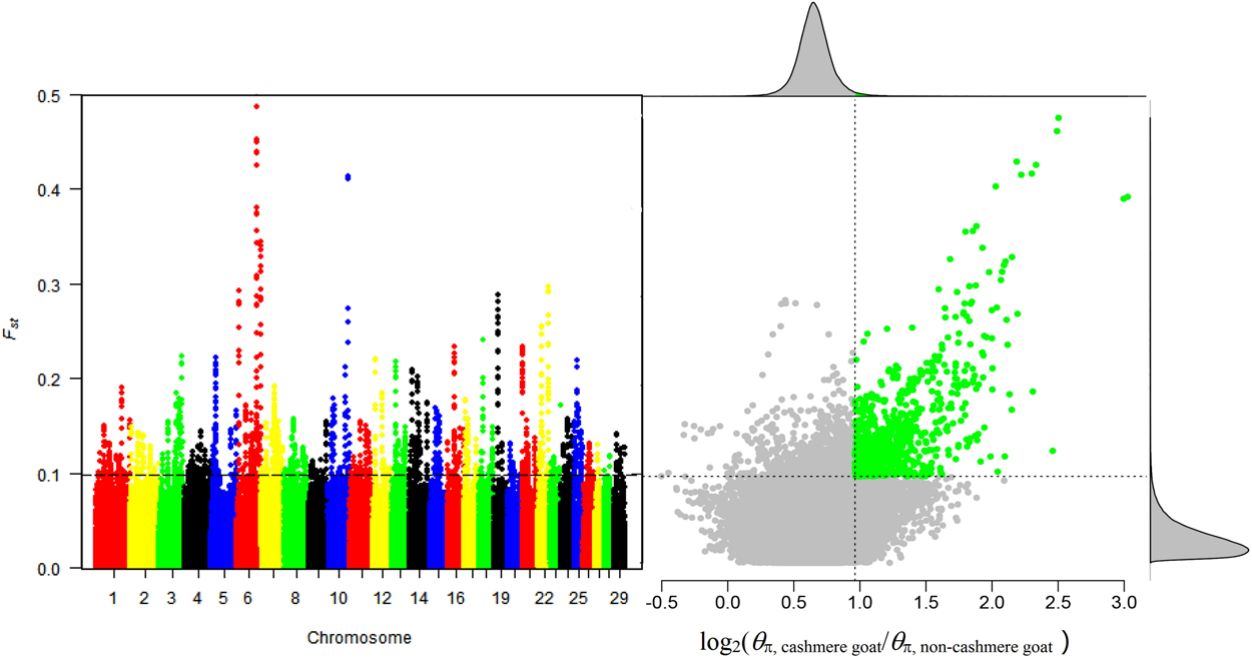
Identification of genomic regions with strong selective sweep signals in Cashmere goats. Distribution of log2(θ_π_ ratio(θ_π, cashmere goat_/θ_π, non-cashmere goat_) and *F*_ST_, which are calculated in 100 kb windows sliding in 10 kb steps. Data points located to the right of the vertical lines (corresponding to 5% right tails of the empirical log2 (θ_π_ ratio) distribution, where log2 (θ_π_ ratio) is 1.26) and above the horizontal line (5% right tail of the empirical *F*_ST_ distribution, where *F*_ST_ is 0.10) were identified as selected regions for cashmere goat (blue points).

**Figure 5:**
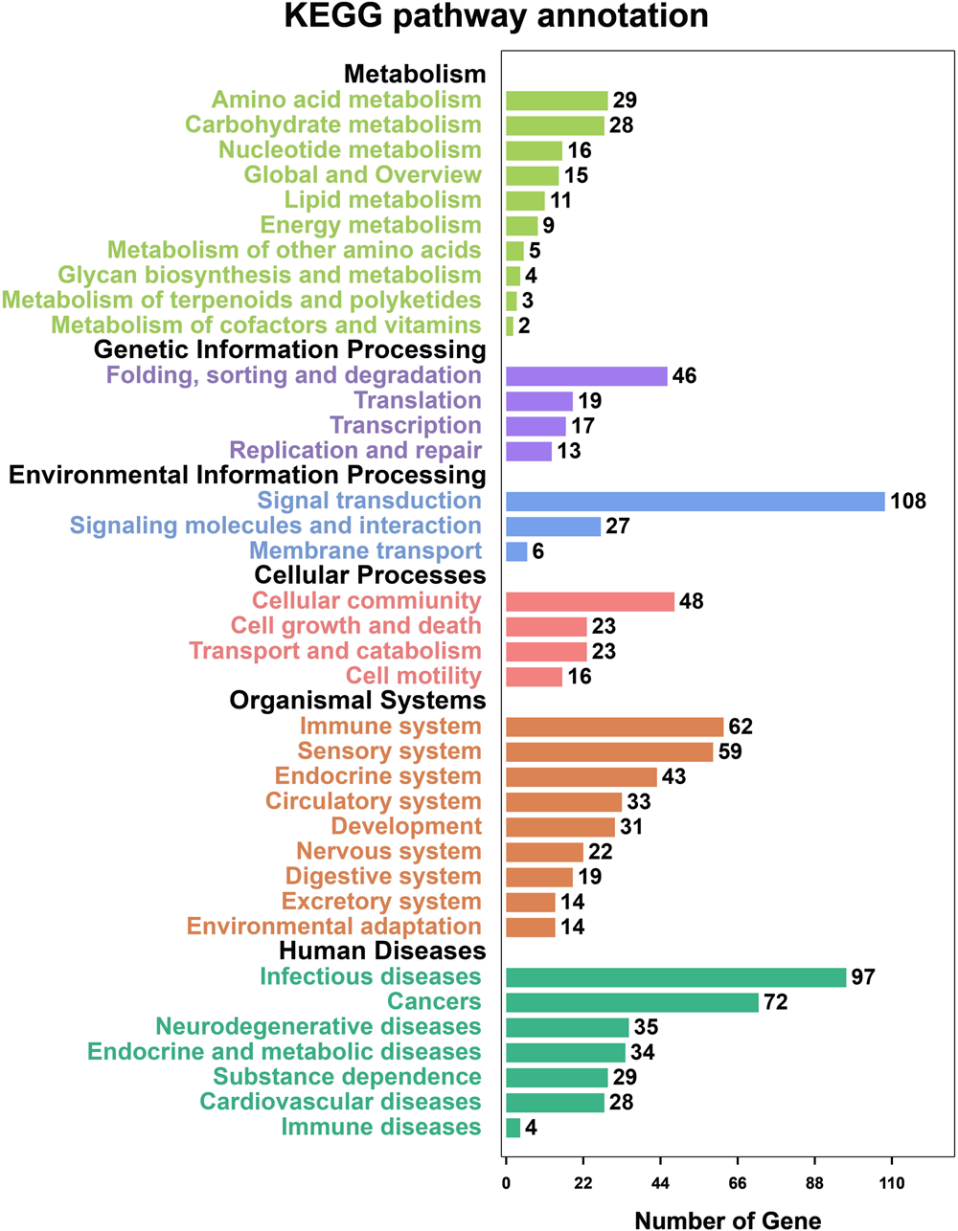
KEGG pathways enrichment analysis of candicate genes under selection within cashmere goats.

In order to detect genome selection signals and SNPs related to cashmere fiber traits, we used 14 unpublished genomic data from non-cashmere goats (~12.50-fold average depth) courtesy of our collaborator (Supplementary Table 6). By using both *θ*_π_ cut-off ratio and high *F*_ST_ values, we identified a total of 135 genomic regions under selective sweep containing 650 candidate genes that were associated with cashmere goat traits (Fig. 4). Gene ontology analysis of these candidate genes revealed enrichments in 206 GO terms in the biological processes, 69 GO terms in the molecular functions, and 25 GO terms in the cellular components with a 5% FDR threshold for significance (Supplementary Data 5). KEGG enrichment analysis of these candidate genes identified 36 pathways with a 5% FDR threshold for significance (Supplementary Data 6 and Fig. 5 **)**. The variant location within selected genes was shown in Supplementary Data 7.

### Candidates genes related to cashmere fiber traits

Candidate genes associated with cashmere fiber traits were identified in several genomic regions under selective sweep, including *ROCK1*, *FGF5*, *PRKCD, SGK3, IGFBP7*, and *OXTR*. *ROCK1* (Rho-associated protein kinase 1) plays an important role in regulation of keratinocyte proliferation and terminal differentiation in human^30,31^. *FGF5* (fibroblast growth factor 5) regulates hair length in many species^32–35^. A previous study showed that disruption of *FGF5* led to more secondary hair follicles and longer fibers in cashmere goat. In a mouse study, overexpression of *PRKCD* (protein kinase C delta) had an inhibitory effect on hair growth. The authors also proposed that *PRKCD* together with *PRKCA* (protein kinase C alpha) kept hair growth in balance^36^. *SGK3* (aliases *SGK2*, serum/glucocorticoid-regulated kinase 3) belongs to the PI3K-Akt signaling pathway, and plays an important role in the development of postnatal hair follicle^37^. Loss of *SGK3* led to reduced proliferation and increased apoptosis of hair follicles in mice^38–40^. Mutations of SGK3 were also responsible for the fuzzy hair phenotype in mice^41^. *OXTR* (oxytocin receptor) is expressed in the primary human dermal fibroblasts and keratinocytes, and *OXT* decreases the proliferation of dermal fibroblasts and keratinocytes in a dose-dependent manner^42^. *IGFBP7* (insulin like growth factor binding protein 7) was found to be one of several keratinocyte-specific genes differentially expressed in keratinocyte^43^.

### Candidate genes related to the adaptation to a cold and dry environment

Cashmere goat usually live in a cold and dry environment. The fine cashmere fibers greatly help these animals to combat heat loss. This adaptive feature is also accompanied by other physiologic mechanisms that help maintain mineral and energy homeostases^23,44^. For example, *ADCY4* (adenylyl cyclase 4) was involved in the regulation of the oxytocin signaling pathway, insulin secretion, adrenergic signaling in cardiomyocytes, rap1 signaling pathway, and cGMP-PKG signaling pathway. Besides, Adenylyl cyclase (AC)‐stimulated cAMP is involved in cAMP‐induced cell proliferation in cultured adrenal cells and a key mediator of Na and water transport^45,46^. Four other genes *ROCK1* (Rho-associated protein kinase 1), *ACNA1C* (calcium voltage-gated channel subunit alpha1 C)*, OXTR* (oxytocin receptor) were also involved in the oxytocin signaling pathway. It was reported that they were all functionally related to the regulation of skin development, fat metabolism, and ion homeostasis. In addition, three candidate genes *CACNA2D1* (calcium channel, voltage-dependent, alpha2/delta subunit 1)*, AGT* (angiotensinogen), and *PTGER2* (prostaglandin E receptor 2) were involved in the renin secretion pathway. *SLC24A4* (Sodium/Potassium/Calcium Exchanger 4) were located in the classical HIF-1 (hypoxia-induced factors) pathway, which plays a central role in regulating cellular responses to hypoxia^19^. *IGFBP7* and *FGF5* have high outlier value indicated under selection, then we further analyzed the allele frequency difference of each SNVs (Supplementary Data 8). It would be interesting to see how the genetic variations in these genes affect the phenotypes of cashmere goat in future studies.

## Conclusion

The use of the latest high-quality reference goat genome assembly provided us more details of the genomic information compared to CHIR_1.0 and CHIR_2.0. To avoid false positives in identifying SNPs and Indels in our study, a series of filtering step were applied to remove low-quality SNPs. This procedure guaranteed high quality genetic variants for the downstream analyses. The large number of genetic variants identified in this study gives us a chance to further explore the genetic diversity and genetic basis of different phenotypes in goats. The population-specific molecular markers can be used to distinguish phenotypically similar animals with higher accuracy.

Our study provided comprehensive insights into the phylogenetic relationship between the two major Chinese cashmere goat breeds. We showed that the Erlangshan cashmere goats were closest related to Liaoning cashmere goats. This genetic information may be useful to explore the domestication and distribution of cashmere goat in Northern China. Our results also provided a large collection of candidate genes that may be targeted for trait improvement. As part of the Hapmap goat project, these cashmere goat genetic footprints and SNPs will serve as a useful tool for the breeding of *Capra* species.

## Methods

### Sample collection

The Inner Mongolia cashmere goat breed was sampled from three independent populations according to their geographical locations: Alashan league, Ordos city, and BayanNur city of Inner Mongolia Autonomous Region. The Liaoning cashmere goat was sampled from one independent population in Gai county of Liaoning province (Fig. 1 and Supplementary Table 1). All four goat populations are raised in local pastures and allowed to free range. With the assistance of local herdsmen, trained veterinarians randomly chose 15–19 three-year-old female goats from each population, and collected 5 ml whole blood from the left jugular vein of each animal into plastic collection tubes containing 4% (w/v) sodium citrate. The blood samples were snap frozen in liquid nitrogen, and stored at −80 °C until delivered to Kunming Institute of Zoology on dry ice for further processing. All experimental procedures were approved by the Animal Care and Use Committee of the Inner Mongolia Agricultural University, and conducted in strict accordance with the animal welfare and ethics guidelines.

### DNA isolation and sequencing

Genomic DNA was extracted from the whole blood samples with the AXYGEN Blood and Tissue Extraction Kit (Corning, USA) according to the manufacturer’s instructions. The extracted DNA were subjected to electrophoresis in 2% agarose gel and stained with ethidium bromide to assess overall quality. The DNA concentration was determined by Quant-iT™ PicoGreen® dsDNA Reagent and Kits (Thermo Fisher Scientific, USA) according to the manufacturer’s instructions. Paired-end libraries with insert size of 300 bp from ~2 μg of sheared genomic DNA were constructed with the procedures of NEB DNA Library Prep Kit for Illumina (NEB, USA). These libraries were sequenced on an Illumina Hiseq. 2000 platform (Illumina; CA, USA) using a PE-101 module. In addition, to characterize the genetic variant relate to cashmere fiber based on selection signals and GWAS, we also downloaded the genome data of 18 individuals from, including.

### Data filtering and clean reads generation

All raw data were first filtered and trimmed using NGSQCToolkit_v2.3.3 if any of the following criteria were met: (1) reads containing adapter and poly-N; (2) low quality reads with >30% bases having Phred quality ≤25; (3) the 5′ and 3′ ends 5 bp low quality base, which is generally considered high bias. This data filtering process resulted in a total of 534.7 Gb clean data from 70 cashmere goats (51 Inner Mongolia breed and 19 Liaoning breed), achieving an average 2.61-fold individual genomic coverage depth. At the population level, the coverage ranged from 41.02 to 50.88 fold for genetic variation detection and downstream analysis (Supplementary Table 1).

### Variation discovery

The clean reads were aligned to the recently released version of the reference goat genome (ARS1)^23,47^ using Burrows-Wheelser Aligner v0.7.10-r789^48^ with default settings. The reference genome sequence was indexed with bwa. The algorithm MEM was used to find the suffix array coordinates of good matches for each read^49^. SAMtools^50^ was used to convert file format from SAM to BAM and to filter the unmapped and non-unique reads. Picard (version 1.106, http://broadinstitute.github.io/picard/) was used to sort the BAM files, and remove potential PCR duplication if multiple read pairs had identical external coordinates. Read pairs with top mapping quality were retained. Local realignment around short insertions and deletions (Indels) was performed with duplication-removed reads using RealignerTargetCreator and IndelRealigner in the Genome Analysis Toolkit (GATK, version 3.3-0-g37228af)^51^. After local realignment, ‘HaplotypeCaller’ in GATK was used for generating a single call set in all individuals by joint calling. Single nucleotide polymorphisms (SNPs) and Indels were separated with the GATK tool ‘selectVariants’, and subjected to rigorous processing to exclude false positives. The SNP exclusion criteria^52^ were as follows: (1) hard filtration with parameter ‘QD < 2.0 || ReadPosRankSum <−8.0 || FS > 10.0 || QUAL < 1349.1’; (2)“–max-missing 0.7 ||–maf 0.05”. The Indel exclusion criteria were as follows: (1) hard filtration with parameter ‘QUAL < 20.0 || QD < 2.0 || ReadPosRankSum <−8.0 || FS > 10.0 || QUAL < 1257.74; (2) “–maxIndelSize 25 ||–maf 0.05”, only insertions and deletions shorter than or equal to 25 bp indels were taken into account. Except Venn diagram, only mapped autosomal SNPs and Indels were included in the downstream analyses.

### Genomic annotation of SNPs and Indels

All filtered SNPs and Indels were annotated and categorized by packages Annovar with default settings^53^. Venn diagrams representing SNVs were generated using the online method (http://bioinformatics.psb.ugent.be/webtools/Venn/). The transition-to-transversion (Ti/Tv) ratio based on all detected SNPs was calculated to evaluate potential sequencing errors, which is used as an indicator of potential sequencing errors^52^. The average ratios of homozygous versus heterozygous and nucleotide diversity are calculated for Inner Mongolia and Liaoning cashmere goat with VCFTools^54^.

### Population structure analysis

To explore their phylogenetic relationship, the whole-genome autosomal SNPs were extracted to construct the phylogenetic tree, and genotypes of sheep sequence were used to provide out-group information at corresponding positions. The neighbor-joining tree was constructed using the PHYLIP 3.696 software (http://evolution.genetics.washington.edu/phylip.html) based on distance matrix methods^55^. iTOL (http://itol.embl.de) was used to illuminate and visualize the phylogenetic tree^56^.

For both of principal component (PCA) and population structure analysis, a thined SNPs dataset with a window of size 50 SNPs advanced by 5 SNPs at a time and an linkage-disequilibrium r2 threshold of 0.5 were filtered using PLINK (Version v1.90b3.38)^57^ PCA was performed with the Genome-wide Complex Trait Analysis (GCTA, version: 1.25.3) software^58^, and the first three eigenvectors (two eigenvectors for PCA analysis of Liaoning and Inner Mongolia) were plotted. Population structure was analyzed using the ADMIXTURE (Version: 1.3)^59^ program which implement a block-relaxation algorithm. To explore the convergence of individuals, we predefined the number of genetic clusters K from 2–6 and ran with cross-validation error (CV) procedure. Default methods and settings were used in Admixture analysis. Population differentiation index (*F*st) was measured by pairwise *F*st values among pariwise populations^54^.

Linkage disequilibrium (LD) was calculated using PLINK software^57^. The pairwise r2 values within and between different chromosomes were calculated. Regarding the LD for overall genome, the r2 value was calculated for individual chromosomes using SNPs from the corresponding chromosome with parameter “–ld-window-r2 0–ld-window 99999–ld-window-kb 1000–r2”, and then the pairwise r2 values were averaged across the whole genome. The LD for each group was calculated using SNP pairs only from the corresponding group.

### Genome scanning for selective signals

We performed a genetic differentiation (*F*st) and polymorphism levels (θπ, pairwise nucleotide variation as a measure of variability) based cross approaches to investigate the selection signals across the whole genome. A 100 kb sliding window with 10 kb step approach was applied to quantify *F*st and θπ, and the cross top 10% of two values was selected as selective signals.

### Functional enrichment analysis (GO and KEGG)

GO and KEGG enrichment analysis was performed using the OmicShare tools, a free online platform for data analysis (www.omicshare.com/tools). Firstly, all candidate genes were mapped to GO terms in the Gene Ontology database (http://www.geneontology.org/), gene numbers were calculated for every term, significantly enriched GO terms in genes comparing to the genome background were defined by hypergeometric test. The calculated p-value was gone through FDR Correction, taking FDR ≤0.05 as a threshold. GO terms meeting this condition were defined as significantly enriched GO terms in candidate genes. Secondly, all candidate genes were mapped to KO terms in the KEGG Pathway database (http://www.genome.jp/kegg/ko.html). KEGG pathway enrichment analysis identified significantly enriched metabolic pathways or signal transduction pathways in candidate genes comparing with the whole genome background. The calculating formula and significantly enriched standard is the same as that in GO analysis.

### Availability and Requirements

The sequencing reads of each sequencing libraries have been deposited under NCBI with Project ID SRP082615.

## Electronic supplementary material

Supplementary Information

Supplementary Dataset 1

Supplementary Dataset 2

Supplementary Dataset 3

Supplementary Dataset 4

Supplementary Dataset 5

Supplementary Dataset 6

Supplementary Dataset 7

Supplementary Dataset 8
