# Supplementary material for: Identification of selection signals by large-scale whole-genome resequencing of cashmere goats
Source: Sci Rep. 2017 Nov 9;7:15142. doi: 10.1038/s41598-017-15516-0 (PMC5680388; doi:10.1038/s41598-017-15516-0)
Supplement: Supplementary file 1 — Supplementary Information [file 41598_2017_15516_MOESM1_ESM.pdf]

Supplementary Information for

Identification of selection signals by large-scale whole-genome resequencing of cashmere goats

Xiaokai Li[1#], Rui Su[1,2,3,4,5#], Wenting Wan[6#], Wenguang Zhang[1], Huaizhi Jiang[7], Xian Qiao[1], Yixing Fan[1], Yanjun Zhang[1,2,3,4], Ruijun Wang[1,2,3,4], Zhihong Liu[1,2,3,4],Zhiying Wang[1,2,3,4], Bin Liu[8], Yuehui Ma[9], Hongping Zhang[10], Qianjun Zhao[9], Tao Zhong[10], Ran Di[9], Yu Jiang[11], Wei Chen[12,14], Wen Wang[5*], Yang Dong[12,13,14*], Jinquan Li[1,2,3,4]

[1] College of Animal Science, Inner Mongolia Agricultural University, Hohhot, Inner Mongolia, China, 010018.

[2] Key Laboratory of Animal Genetics, Breeding and Reproduction - Inner Mongolia Autonomous Region, Inner Mongolia Agricultural University, Hohhot, Inner Mongolia, China, 010018.

[3] Key Laboratory of Mutton Sheep Genetics and Breeding, Ministry of Agriculture, Inner Mongolia Agricultural University, Hohhot, Inner Mongolia, China, 010018.

[4] Engineering Research Center for Goat Genetics and Breeding - Inner Mongolia Autonomous Region, Inner Mongolia Agricultural University, Hohhot, Inner Mongolia, China, 010018.

[5] State Key Laboratory of Genetic Resources and Evolution, Kunming Institute of Zoology, Chinese Academy of Sciences, Kunming, Yunnan, China, 650223.

[6] Center for Ecological and Environmental Sciences, Key Laboratory for Space Bioscience & Biotechnology, Northwestern Polytechnical University, Xi'an, Shaanxi, China, 710072.

[7] College of Animal Science and Technology, Jilin Agricultural University, Changchun, Jilin, China, 130118.

[8] Institute of Animal Husbandry, Academy of Agriculture and Stockbreeding Sciences, Hohhot, Inner Mongolia, China, 010030.

[9] The Key Laboratory for Farm Animal Genetic Resources and Utilization of Ministry of Agriculture of China, Institute of Animal Science, Chinese Academy of Agricultural Sciences, Beijing, China, 100193.

[10] Farm Animal Genetic Resources Exploration and Innovation Key Laboratory of Sichuan Province, College of Animal Science and Technology, Sichuan Agricultural University, Chengdu, China, 611130.

[11] College of Animal Science and Technology, Northwest A&F University, Yangling, China, 712100.

[12] College of Biological Big Data, Yunnan Agriculture University, Kunming, Yunnan, China, 650504.

[13] BGI-Shenzhen, Shenzhen, Guangdong, China, 518083.

[14] Yunnan Research Institute for Local Plateau Agriculture and Industry, Kunming, Yunnan, China, 650201.

[#]These authors contributed equally to this manuscript.

* To whom correspondence should be addressed:

Wen Wang: wwang@mail.kiz.ac.cn;

Yang Dong: loyalyang@163.com.

Jinquan Li: lijinquan_nd@126.com.

Table of contents

Supplementary Fig. 1-4

Supplementary Fig. 1 | The distribution of site depth of SNP among cashmere goats

Supplementary Fig. 2 | Nucleotide diversity of Liaoning and Inner Mongolia cashmere goats

Supplementary Fig. 3 | GO annotation of Genes with affected SNPs

Supplementary Fig. 4 | GO annotation of Genes with affected Indels

Supplementary Tables 1-6

Supplementary Table 1 | Summary and mapping statistics of cashmere goat.

Supplementary Table 2 | Distribution of SNPs in the cashmere goat genome.

Supplementary Table 3 | Description of heterozygous-to-homozygous (Het/Hom), transition-to-transversion (Ti/Tv) ratios and genetic diversity (π)for cashmere goat with SNVs.

Supplementary Table 4 | Summary and annotation of InDels in cashmere goat.

Supplementary Table 5 | Population relationship of cashmere goat based on *F*st index.

Supplementary Table 6 | Summary and mapping statistics of the non-cashmere goat whole genome resequencing data.

Supplementary Data 1-8

Supplementary Data 1 | Annotation of affected SNPs in cashmere goat. **(see Excel file ‘Supplementary Data 1.xlsx’)**

Supplementary Data 2 | GO annotation of genes with affected SNPs. **(see Excel file ‘Supplementary Data 2.xlsx’)**

Supplementary Data 3 | Annotation of affected Indels in cashmere goat. **(see Excel file ‘Supplementary Data 3.xlsx’)**

Supplementary Data 4 | GO Annotation of genes with affected Indels. **(see Excel file ‘Supplementary Data 4.xlsx’)**

Supplementary Data 5 | GO annotation of functional genes under selection for cashmere goats. **(see Excel file ‘Supplementary Data 5.xlsx’)**

Supplementary Data 6 | KEGG pathway analysis of functional genes under selection for cashmere goats. **(see Excel file 'Supplementary Data 6.xlsx')**

Supplementary Data 7 | The variant location within selected genes. (**see Excel file 'Supplementary Data 7.xlsx')**

Supplementary Data 8 | Allele frequency difference of SNV sites within *IGFBP7* and *FGF5*. (**see Excel file 'Supplementary Data 8.xlsx')**

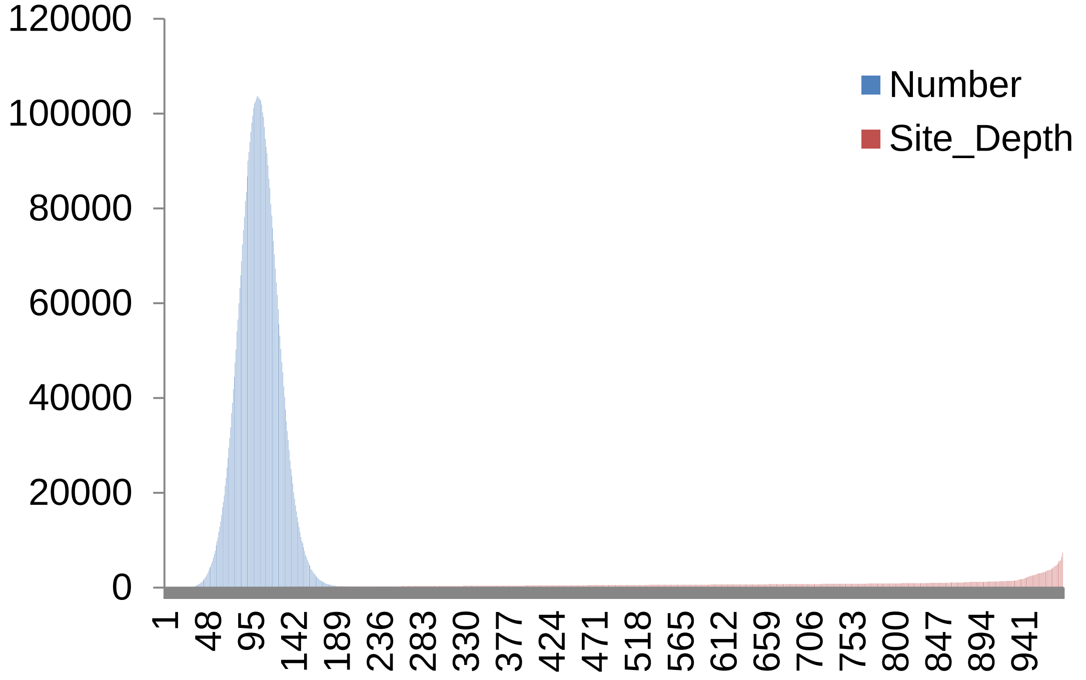

Supplementary Fig. 1 | The distribution of site depth of SNPs among cashmere goats

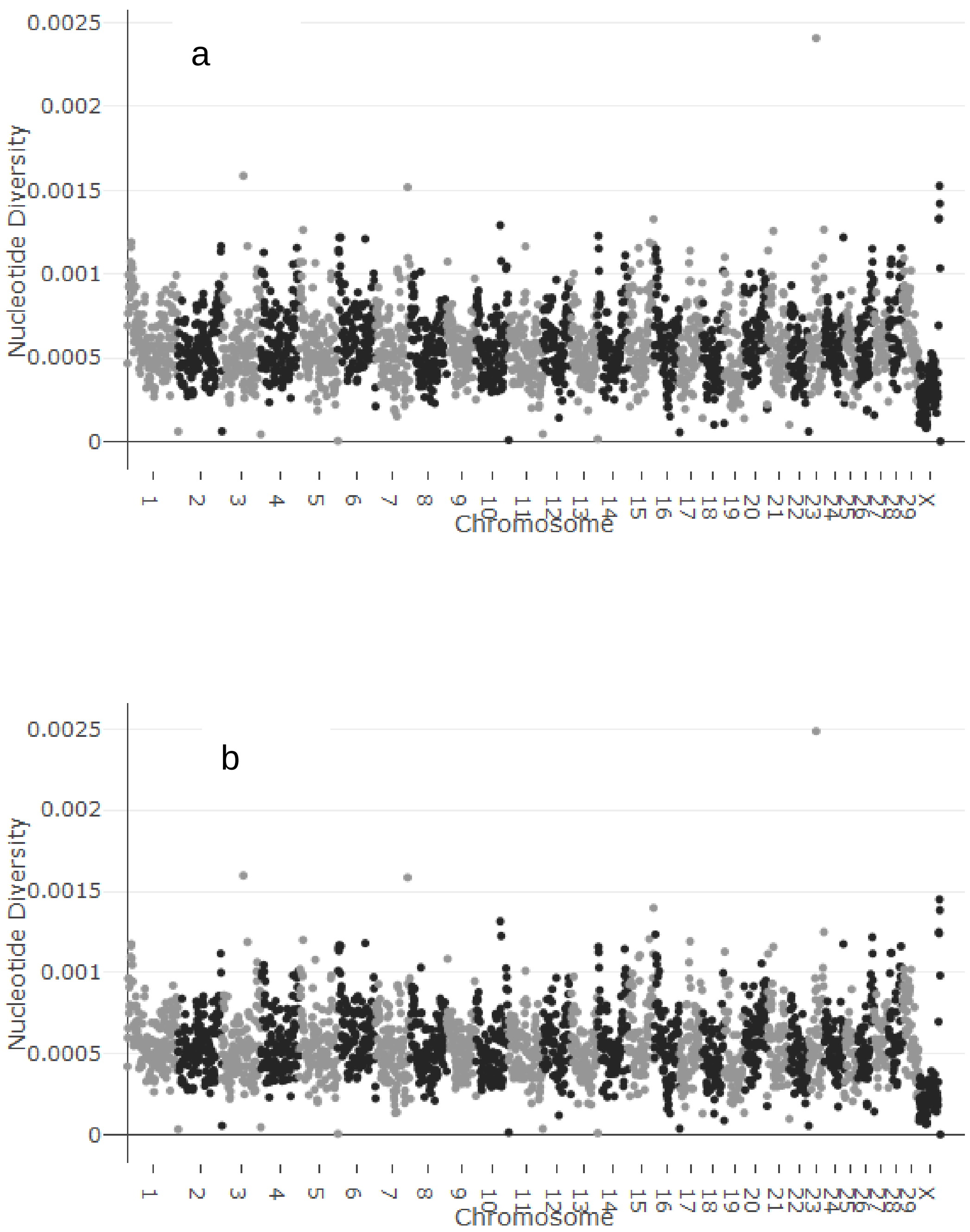

Supplementary Figure 2 | Nucleotide diversity of Liaoning and Inner Mongolia cashmere goat. Nucleotide diversity of 10^6-bp nonoverlapping window is shown. **a.** Nucleotide diversity for Inner Mongolia cashmere goat. **b.** Nucleotide diversity for Liaoning cashmere goat.

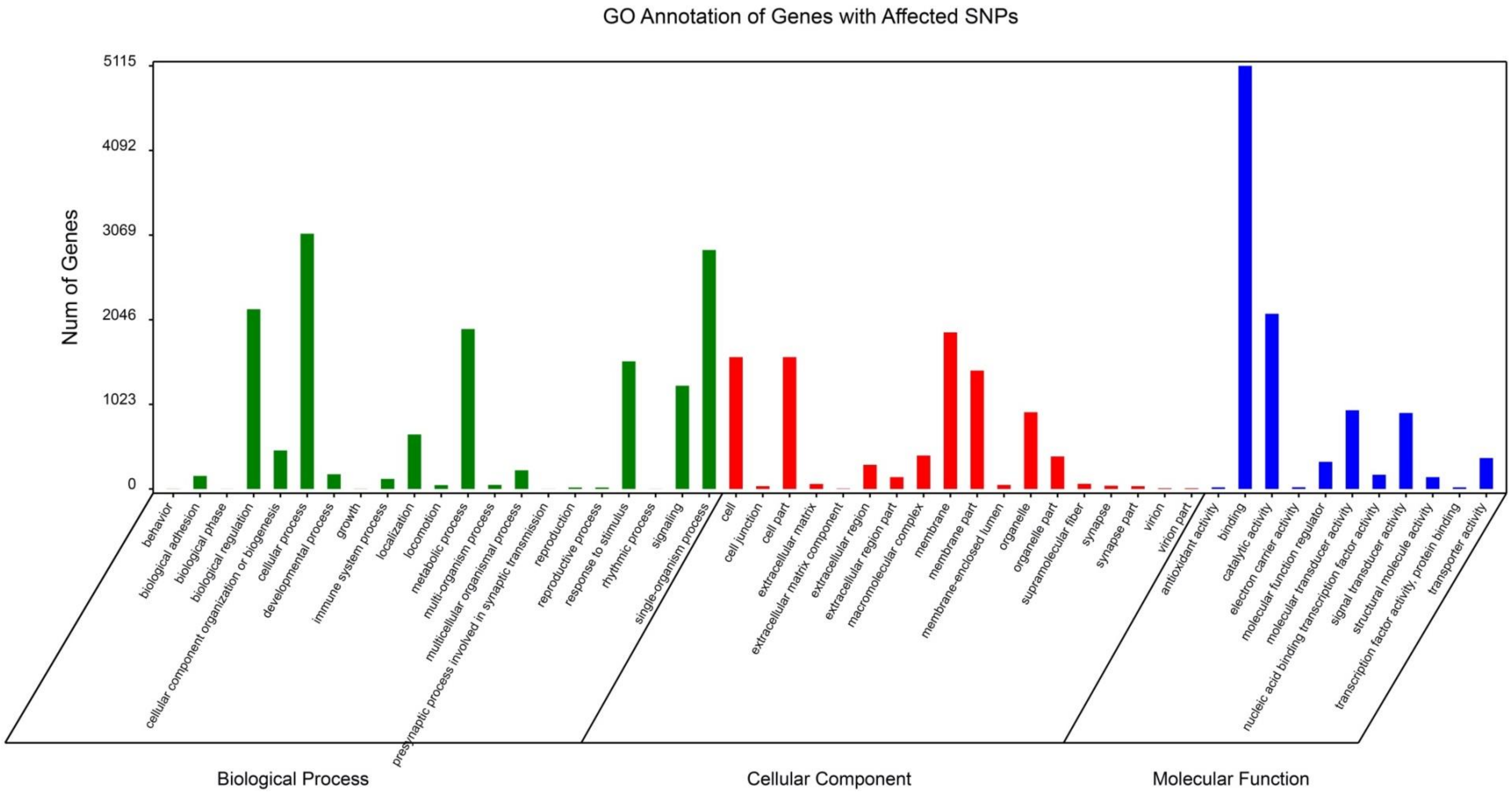

Supplementary Fig. 3 | GO annotation of Genes with affected SNPs.

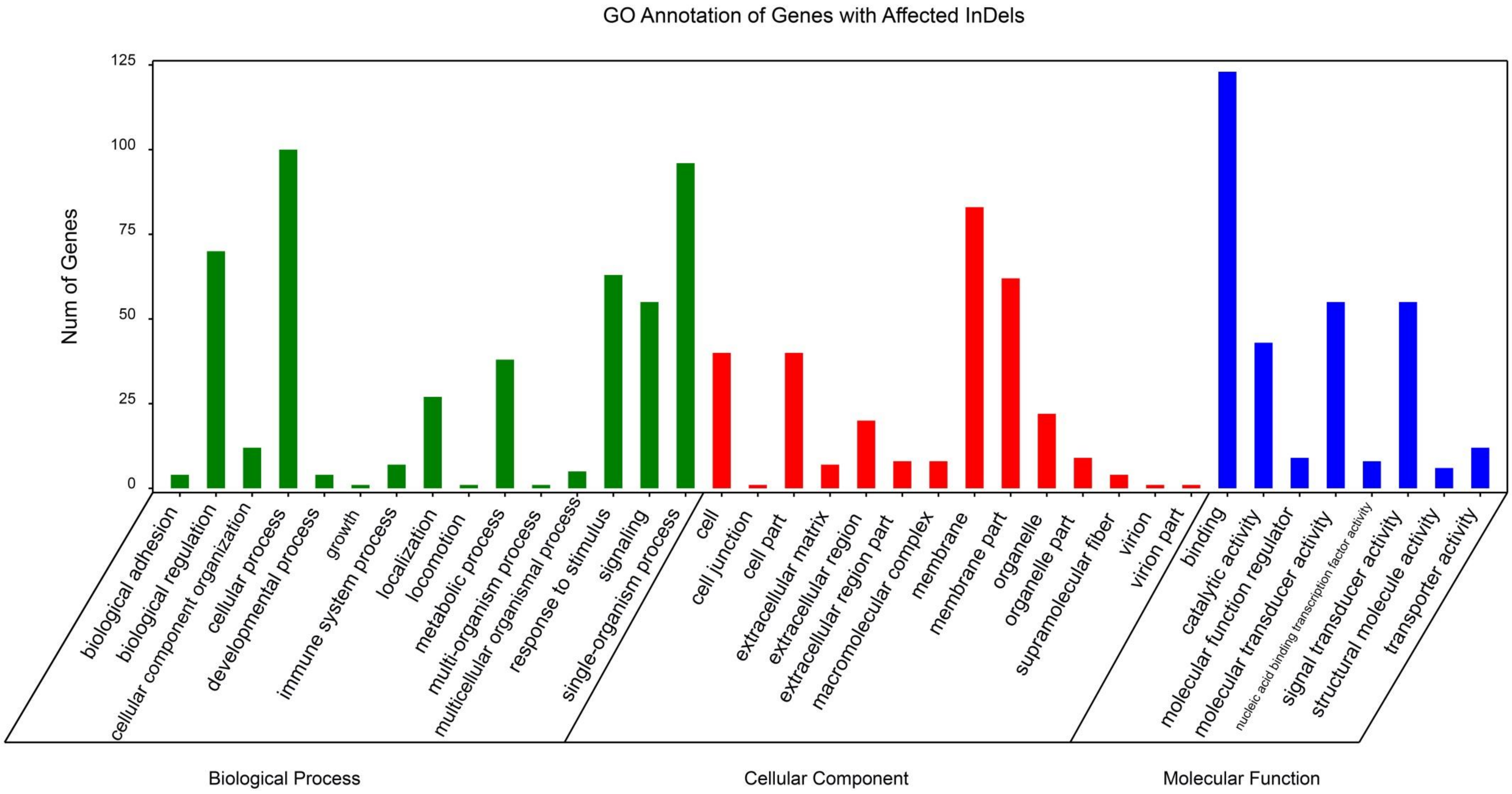

Supplementary Fig. 4 | GO annotation of Genes with affected Indels.

Supplementary Table 1 | Summary and mapping statistics of cashmere goat.

Population	Location	Sample No.	PE length (bp)	Raw base (Gb)	High-quality base (Gb)	Mapping rate (%)	Total Depth (×)	Average Depth (×)
Aerbasi	Erdos City, Inner Mongolia	15	101	139.13	119.91	99.92	41.03	2.74
Alashan	Alxa League, Inner Mongolia	17	101	141,47	121.99	99.80	41.74	2.46
Erlangshan	Bayannur League, Inner Mongolia	19	101	165.54	144.04	99.81	49.28	2.59
Liaoning	Gaizhou City, Liaoning	19	101	165.53	148.72	99.86	50.88	2.68

*Aerbasi, Alashan and Erlangshan were represented three geographical isolated independent populations of Inner Mongolia cashmere goats.

Supplementary Table 2 | Distribution of SNPs in the cashmere goat genome.

Chr	Physical size (Mb)	SNP no. (after filtered)	Kb/SNP (after filtered)	SNP density SNP/Kb
1	157.40	363508	0.43	2.31
2	136.51	286401	0.48	2.10
3	120.04	245380	0.49	2.04
4	120.47	273052	0.44	2.27
5	119.02	252609	0.47	2.12
6	117.64	289666	0.41	2.46
7	108.43	228654	0.47	2.11
8	112.67	238540	0.47	2.12
9	91.57	193300	0.47	2.11
10	101.09	217661	0.49	2.05
11	106.23	207173	0.51	1.95
12	87.28	197292	0.44	2.26
13	83.03	163065	0.51	1.96
14	94.67	211276	0.45	2.23
15	81.90	196025	0.42	2.39
16	79.37	171720	0.46	2.16
17	71.14	155516	0.46	2.19
18	67.28	123451	0.54	1.83
19	62.52	114081	0.55	1.82
20	71.78	169902	0.42	2.37
21	69.43	150446	0.46	2.17
22	60.28	114207	0.53	1.89
23	48.87	118327	0.41	2.42
24	62.31	135804	0.46	2.18
25	42.86	82693	0.52	1.93

26	51.42	117061	0.44	2.28
27	44.71-	97815	0.46	2.19
28	44.67	114498	0.39	2.56
29	51.33	123189	0.42	2.40
X	115.94	147329	0.79	1.27
Sum	2581.86	5489641	0.48*	2.14*

* indicates the mean distance of SNPs in goat chromosomes.The filtered SNP means that qualified SNP also meet the filtering criterion MAF >0.05.

Supplementary Table S3 | Description of heterozygous-to-homozygous (Het/Hom) and transition-to-transversion (Ti/Tv) ratios and genetic diversity (π) for cashmere goat with SNVs.

	Het/Hom	Pi (10^{-4})	Ts/tv
**Aerbasi**	3.50	5.83	2.36
**Alashan**	3.82	5.31	2.36
**Erlangshan**	4.06	5.76	2.36
**Liaoning**	3.78	5.63	2.36

Supplementary Table S4 | Summary and annotation of InDels in cashmere goat

Category		Number of InDels	Percent(%)
3’UTR		4,460	0.63
5’UTR		858	0.12
Downstream		4,954	0.69
Exonic/	Frameshift deletion	131	
	Frameshift insertion	153	
	Nonframeshift deletion	143	
	Nonframeshift insertion	145	0.09
	Stop gain	14	
	Stop loss	1	
	Unkown	62	
Intergenic		450,677	63.42
Intronic		243,681	34.29
NcRNA_5’UTR		1	0
NcRNA_exonic		111	0.02
NcRNA_intronic		314	0
Splicing		42	0
Upstream		4713	0.66
Upstream/Dowstrean		140	0.02
Total		710600	

Supplementary Table S5 | Population relationship of cashmere goat based on *F*st index.

	Aerbasi	Alashan	Erlangshan
Aerbasi			
Alashan	0.0395937		
Erlangshan	0.0431378	0.0282739	
Liaoning	0.110202	0.0999503	0.0569453

Supplementary Table S6 | Summary and mapping statistics of the non-cashmere goat whole genome resequencing data.

Breed	Land of origin	PE length (bp)	Raw base (Gb)	High-quality base (Gb)	Mapping rate (%)	Depth (×)
Alpine	France	100	46.01	39.88	99.91	13.64
Boer	South African	100	48.99	42.72	99.93	14.62
Chengdu Brown goat	Sichuan Province, China	100	44.90	36.90	99.91	12.63
Creole	Agentine	100	45.47	34.98	99.79	11.97
Leizhou goat	Guangdong Province, China	100	43.73	40.04	99.89	13.70
Huanghai goat	Anhui Province, China	100	45.15	36.80	99.79	12.59
Guizhou Black goat	Guizhou Province, China	100	46.17	38.77	99.64	13.26
Yaoshan White goat	Henan Province, China	100	46.02	37.83	99.66	12.94
Jianchang Black goat	Sichuan Province, China	100	36.12	34.00	99.89	11.63
Matou goat	Hubei Province, China	100	44.50	38.83	99.91	13.29
Yudong white goat	Chongqi City, China	100	41.52	27.97	99.88	9.5
Banjiao goat	Sichuan Province,China	100	43.23	38.10	99.85	13.04
Maguan Poll goat	Yunnan Province, China	100	31.49	29.70	99.91	10.16
Guishan goat	Yunnan Province, China	100	37.63	35.21	99.91	12.05
